# Effect of Complex Well Conditions on the Swelling and Tribological Properties of High-Acrylonitrile Stator Rubber in Screw Pumps

**DOI:** 10.3390/polym16142036

**Published:** 2024-07-17

**Authors:** Xinfu Liu, Xiangzhi Shi, Zhongxian Hao, Songbo Wei, Yi Sun, Xinglong Niu, Chunhua Liu, Ming Li, Zunzhao Li

**Affiliations:** 1 Key Lab of Industrial Fluid Energy Conservation and Pollution Control (Ministry of Education), Qingdao University of Technology, Qingdao 266520, China; xzshi1999@163.com (X.S.);; 2 Research Institute of Exploration & Development, PetroChina, Beijing 100083, China; 3 College of Mechanical and Electronic Engineering, China University of Petroleum (East China), Qingdao 266580, China; 4 SINOPEC DaLian Research Institute of Petroleum and Petrochemicals, Dalian 116045, China

**Keywords:** complex well conditions, high-acrylonitrile stator rubber, screw pump, thermal expansion and swelling, friction and wear

## Abstract

The effects of complex well conditions in shale oil wells on the swelling and tribological properties of high-acrylonitrile stator rubber used in screw pumps were investigated in this study. Tests were conducted considering the combined effects of immersion medium, temperature, and duration. The key parameters measured included mass change rate, volume change rate, hardness, elongation at break, tensile strength, surface micro-morphology of the rubber after thermal expansion and swelling, friction coefficient, and wear quantity. The results indicated that in the actual well fluids, the mass change rate of high-acrylonitrile rubber ranged from −1.08% to 1.29%, with a maximum volume change rate of 2.78%. In diesel oil, the greatest mass change rate of the rubber was 4.68%, and the volume change rate did not exceed ±1%, indicating superior swelling resistance. In both actual well fluids and diesel oil, the maximum decreases in hardness were 8.7% and 9.5%, respectively. Tensile strength and elongation at break decreased with increasing immersion temperature, with elongation at break in 80 °C diesel oil decreasing by over 50%, indicating a significant decline in the tensile properties of the rubber. The average friction coefficient of rubber specimens immersed in actual well fluids at three temperatures, as well as in diesel oil at 25 and 50 °C, decreased compared with the high-acrylonitrile rubber without thermal expansion and swelling. However, the average friction coefficient of rubber specimens immersed in diesel oil at 80 °C increased. The wear quantity of the rubber increased following immersion in both media. Additionally, the friction coefficient and wear quantity of the rubber increased with increasing immersion temperatures. The results of the study can offer valuable insights into assessing the durability of properties in high-acrylonitrile stator rubber under complex well conditions.

## 1. Introduction

Screw pumps are extensively utilized in major oilfields both domestically and internationally due to their numerous advantages, including simple structure, compact size, low investment, high effectiveness, and remarkable adaptability to harsh environments [1,2,3,4,5]. These pumps are commonly employed in high-temperature and high-pressure settings, handling high-viscosity and corrosive liquids containing sand particles [6,7,8,9]. In such complex well environments, the rubber stator undergoes prolonged cyclic extrusion and friction from the rotor, resulting in high-temperature swelling, severe aging, and wear failure. This results in a reduced service life for the screw pump and decreased oil recovery efficiency [10,11,12]. Therefore, the rubber used for the stator of oil recovery screw pumps must exhibit enhanced resistance to swelling, aging, and abrasion [13,14,15,16].

**Figure 1:**
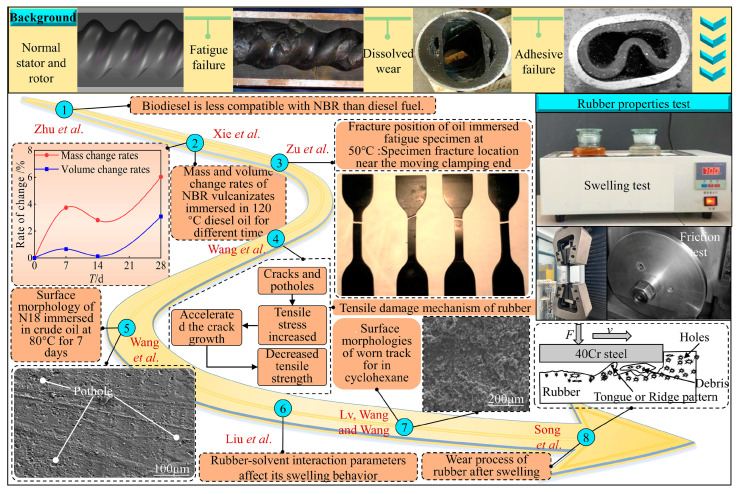
Research status of rubber properties of screw pump stators [24,25,27,28,29,30,31,32,33].

Several studies have focused on elucidating the swelling, aging, and wear properties of rubber (Figure 1). Factors such as the medium, temperature, and type as well as formulation of rubber, significantly influenced these properties [17,18,19,20,21,22,23]. Zhu et al. investigated the compatibility of nine fuels with nitrile butadiene rubber (NBR), and the soaking test results showed that diesel oil had less impact on the quality, volume, and mechanical properties of NBR than biodiesel [24]. Liu et al. studied the swelling behavior of NBR in seven different types of organic solvents. The results showed that the swelling behavior of NBR was related to the solubility parameter of the solvent, and the swelling value increased with the increase in the Flory–Huggins interaction parameter [25]. Cadambi et al. studied the effect of organic clay reinforcers on the properties of HNBR after long-term oxidative aging. The results showed that the swelling and mechanical properties of hydrogenated nitrile butadiene rubber/nano-clay nanocomposites were improved in aliphatic oil and aromatic oil [26].

Notably, temperature exerts a significant impact on the physical properties and wear mechanisms of rubber [34,35]. Wang et al. studied the mechanical properties of rubber before and after swelling in asphalt at different temperatures through experiments. The results showed that the rubber swelling was fast in the early stage, and then, the swelling slowed down. In addition, the aliphatic compounds in the asphalt first diffuse into the rubber during the swelling process [36]. Wang et al. examined the effect of different temperatures of crude oil on the mechanical and tribological properties of NBR, and the results showed that as immersion temperature rises, rubber tensile strength falls and abrasion increases [27]. Qi et al. investigated the friction properties of PTFE/FVMQ/MVQ composites at different temperatures. Compared with silicone rubber, the mechanical properties of PTFE/FVMQ/MVQ composites are basically unchanged, and the friction coefficient is almost unaffected by temperature. The high-temperature wear mode is mainly adhesive wear [37]. Shi et al. studied the mechanical properties of rubber bushing at different temperatures through a uniaxial tensile test and fatigue testing. It was found that long-term aging reduced the hardness, tensile strength, and elongation of the material [38]. Persson et al. discovered that rubber abrasion increased with an increase in temperature [39]. Zu et al. conducted unidirectional tensile and fatigue tests of stator rubber under the 50 °C oil immersion condition. The fatigue life curve in the range of large elongation ratio was obtained. The characteristic parameters of the fatigue life prediction model were determined [28]. Consequently, it is crucial to investigate the influence of various temperature conditions on the performance of screw pump stator rubber.

Additionally, some scholars have studied the properties of different formulations and types of rubber. Lv et al. studied the effect of swelling in cyclohexane solution on the aging and wear properties of N18 and N41 nitrile rubber with acrylonitrile contents of 18% and 41% (N18 and N41). The results showed that N41 had excellent swelling resistance, aging resistance, and wear resistance compared with N18 [29]. Qian et al. investigated the thermal oxygen aging and mechanical tribological properties of nitrile rubber composites with acrylonitrile contents of 28%, 33%, and 41% through molecular dynamics simulation. The results showed that the properties of NBR composites with 41% acrylonitrile content were better [40]. Wang et al. examined the impact of varying acrylonitrile concentrations on the friction properties of NBR after aging. They found that the quantity of abrasion decreases as the acrylonitrile content increases [30]. Song et al. concluded that the wear of NBR decreased with the increase in acrylonitrile content after swelling [31,32]. The stator rubber friction test was carried out under the same conditions, and the wear amount of the fluorine rubber surface was less than that of the nitrile rubber surface [33,41]. It can be seen that an appropriate increase in acrylonitrile content can improve the wear resistance of rubber.

In summary, although there is much research on rubber at present, there is a lack of studies on the influence of complex well conditions in shale oil wells on the properties of high-acrylonitrile stator rubber used in screw pumps. In addition, there is little research on rubber properties that carry out thermal expansion and swelling tests, as well as friction and wear tests at the same time. Therefore, this study utilized high-acrylonitrile rubber as the test material for screw pump stator rubber, which was distinct from the usual nitrile stator rubber used in the oilfield, and comprehensively considered factors such as different immersion media, different temperatures, and different durations. The study conducted thermal expansion and swelling tests, as well as friction and wear tests on the high-acrylonitrile stator rubber of screw pumps to determine the mass change rate, volume change rate, hardness, elongation at break, tensile strength, surface micro-morphology, surface friction coefficient, and wear quantity of the rubber. The effect of complex well conditions on the swelling and tribological properties of high-acrylonitrile stator rubber in screw pumps was studied. This study can provide a reference for evaluating the performance of high-acrylonitrile stator rubber in screw pumps in the complex well environment of shale oil wells. It is of great significance for the selection of stator rubber in the oil field, the extension of the service life of screw pumps, and the improvement in oil production efficiency [42,43].

## 2. Experiments

### 2.1. Selection of Rubber Materials and Test Medium

For the test, 2 mm thick high-acrylonitrile rubber was used, with specimen dimensions of 60 mm × 20 mm × 2 mm. The rubber had been treated with a vulcanization process. The fluid characteristics of shale oil well fluids are relatively complex, with gases such as carbon dioxide, hydrogen sulfide, methane, and other corrosive media in crude oil potentially invading the stator rubber under high temperature and pressure, thereby affecting its performance. To investigate this impact, the actual well fluid from shale oil wells in an oil field and No. 0 diesel oil (referred to as diesel oil) were selected as the test media for thermal expansion and swelling tests.

### 2.2. Major Equipment and Instruments

**Figure 2:**
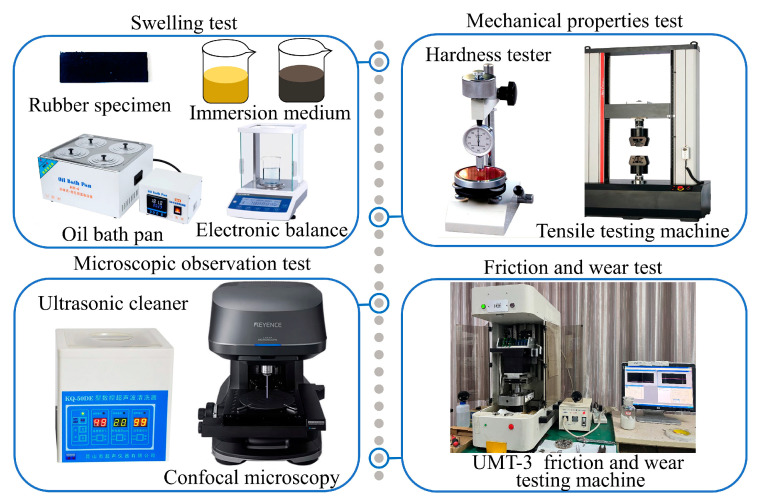
Instruments and equipment employed in the test trials.

The equipment and instruments employed in the test include an HH-4T digital display electric constant temperature oil bath, Shenzhen Dingxinyi Instrument Company Ltd., Shenzhen, China; an FA324 electronic analytical balance, accuracy 0.1 mg, Shanghai Lichen Instrument Technology Company Ltd., Shanghai, China; an LX-A Shore A hardness tester, Shanghai Gaozhi Precision Instrument Company Ltd., Shanghai, China; a WDW-50 computer servo control high-temperature tensile testing machine, Changchun New Test Machine Limited Liability Company, Changchun, China; a KQ-50DE CNC ultrasonic cleaner, Kunshan City Ultrasonic Instrument Company Ltd., Kunshan, China; a VK-X1050 confocal laser microscope, Japan KEYENCE; and a UMT-3 universal friction and wear tester, U.S. CETR products. The instrumentation used for the test is shown in Figure 2.

### 2.3. Test Method

**Figure 3:**
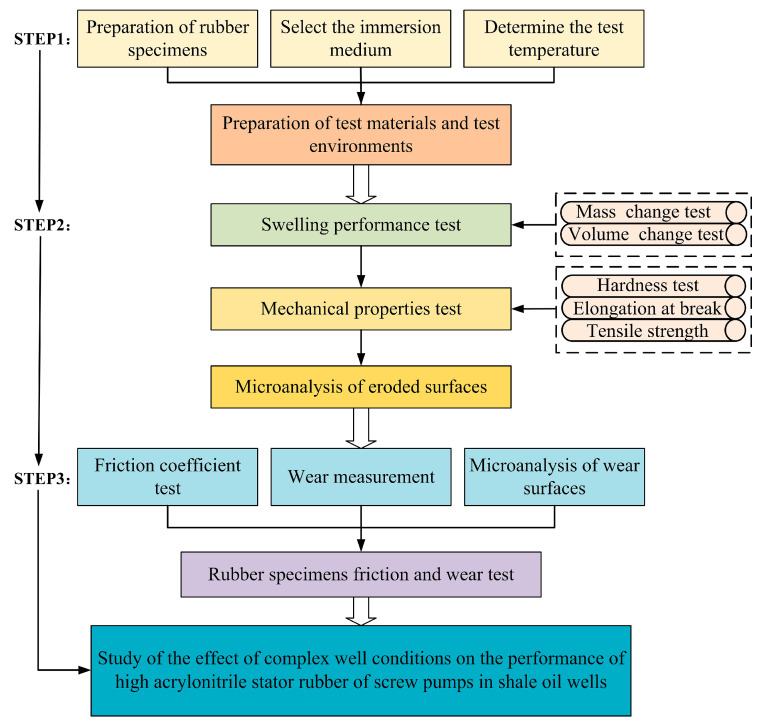
Procedure and analysis of experimental tests and results.

For each series of tests, five rubber specimens were selected. The test results were determined by calculating the median of the hardness test as well as the average of the mass and volume change rate, elongation at break, tensile strength, and wear quantity test of the five specimens. Referring to GB/T 2941-2006 [44], three temperatures of 25 °C, 50 °C, and 80 °C were selected for the thermal expansion and swelling tests of high-acrylonitrile rubber at atmospheric pressure, and the test interval was 10 days, which was conducted for 40 days. After these tests, the friction and wear tests were conducted on the rubber specimens. Figure 3 illustrates the flow of the test trials and analyses.

#### 2.3.1. Swelling Properties Test

The mass and volume change rates of rubber specimens after immersion in actual well fluid and diesel oil were measured. The test method referred to GB/T 1690-2010 [45]. The mass change rate (*R*_M_) of the rubber specimen before and after the immersion was calculated using Equation (1), and the volume change rate (*R*_V_) of the rubber specimen before and after the immersion was calculated using Equation (2).

(1) $$R_{M}=\frac{M_{\mathrm{tA}}-M_{\mathrm{OA}}}{M_{\mathrm{OA}}}\times 100\%$$

(2) $$R_{V}=\frac{\left(M_{\mathrm{tA}}-M_{\mathrm{tW}})-(M_{\mathrm{OA}}-M_{\mathrm{OW}}\right)}{M_{\mathrm{OA}}-M_{\mathrm{OW}}}\times 100\%$$

where *M*_OA_ is the mass of rubber in the air before immersion (mg), *M*_tA_ is the mass of rubber in the air after immersion (mg), *M*_OW_ is the mass of rubber in deionized water before immersion (mg), and *M*_tW_ is the mass of rubber in deionized water after immersion (mg). *M*_OW_ is the mass of the rubber in deionized water before immersion (mg), and *M*_tW_ is the mass of the rubber in deionized water after immersion (mg).

#### 2.3.2. Mechanical Property Test

The hardness, elongation at break, and tensile strength of rubber specimens after immersion in actual well fluid and diesel oil were measured. The test method referred to GB/T 531.1-2008 [46] and GB/T 528-2009 [47]. The hardness test result is accurate to 0.1, and the hardness change (*R*_H_) of the rubber specimen before and after immersion was calculated using Equation (3):

(3) $$R_{H}=H_{t}-H_{0}$$

where *H*_t_ is the hardness of the rubber specimen after immersion (HA) and *H*_0_ is the initial hardness of the rubber specimen (HA).

The elongation at break (*R*_E_) was calculated using Equation (4):

(4) $$R_{E}=\frac{L_{t}-L_{0}}{L_{0}}\times 100\%$$

where *L*_t_ is the test length of the rubber specimen at tearing (mm) and *L*_0_ is the initial test length of the rubber specimen (mm).

The tensile strength (*R*_TS_) was calculated using Equation (5):

(5) $$R_{\mathrm{TS}}=\frac{F_{m}}{W_{h}}$$

where *F*_m_ is the maximum tensile force recorded in the test (N), *W* is the width of the rubber specimen (mm), and *h* is the thickness of the rubber specimen (mm).

#### 2.3.3. Microscopic Observation and Analysis

The surface micro-morphology of the rubber specimens after thermal expansion, swelling, surface wear after friction, and wear tests were observed. After these tests were conducted, the rubber specimens were cleaned in an ultrasonic cleaner using acetone, anhydrous ethanol, and deionized water. Thus, the cleaning time was set at 10 min. Subsequently, following the cleaning process, the specimens were dried using filter paper. The erosion of the rubber surfaces and the wear of the surfaces were examined using a confocal laser microscope (VK-X1050 confocal laser microscope, Osaka, Japan, KEYENCE).

#### 2.3.4. Friction and Wear Property Test

Friction and wear tests were conducted on the rubber specimens following the thermal expansion and swelling examinations to determine the surface friction coefficient and wear quantity. The decrease in hardness of the rubber specimens after these tests affected the wear resistance of the rubber. It was observed from Equation (6) that hardness decreased with increasing wear quantity [48].

(6) $$R_{W}=K\frac{N_{vt1}}{H}$$

where *K* is the wear coefficient; *N* is the normal load (N); *v* is the wear speed (m/s); *t*_1_ is the wear time (s); and *H* is the hardness of the rubber specimen, HA.

**Figure 4:**
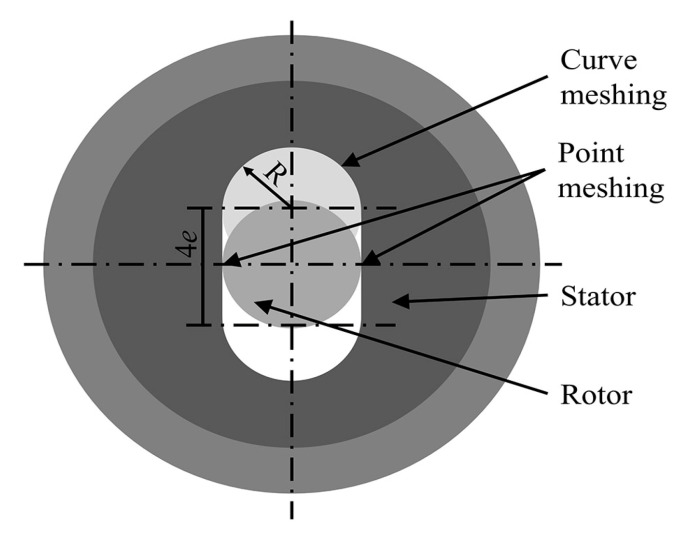
Single-screw pump rotor movement trajectory.

The wear degree of the stator rubber in the oil recovery screw pump was correlated with the rotor speed. Therefore, it was essential to determine the speed at which the stator and rotor of the screw pump operated relative to each other. Figure 4 shows the movement trajectory of the single-screw pump rotor. The point engagement speed (*v_x_*_1_ and *v_y_*_1_), the line engagement point speed ($\nu {'}_{x1}$ and $\nu {'}_{y1}$), and the maximum speed (*v*_max_), as well as the minimum speed (*v*_min_) of the engagement point were calculated using Equations (7)–(9).

(7) $$\left\{\begin{array}{l} \nu_{x1}=R\omega -2e\omega \sin\omega t\\ \nu_{y1}=-R\omega -2e\omega \sin\omega t \end{array}\right.$$

(8) $$\left\{\begin{array}{l} \nu {'}_{x1}=R\omega \\ \nu {'}_{y1}=-R\omega \end{array}\right.$$

(9) $$\left\{\begin{array}{c} \nu_{\max}=R\omega +2e\omega \\ \nu_{\min}=R\omega -2e\omega \end{array}\right.$$

where *R* is the radius of the semicircle (m), *e* is the eccentricity distance (m), *ω* is the angular velocity of the rotor (rad/s), and *t* is the rotation time of the rotor (s).

For the study of the tribological properties of screw pump stator rubber, some scholars have used ring/block micro-controlled wear testing machines to simulate the reciprocating motion of stator rubber in application [31,32,33,41] and the reciprocating motion of steel balls on the surface of rubber specimens. The interference between the stator and rotor of the screw pump was considered. In order to be more consistent with the reciprocating motion of stator rubber in application, the reciprocating motion of a steel ball on the surface of the rubber specimen after thermal expansion and swelling was adopted in this study.

**Figure 5:**
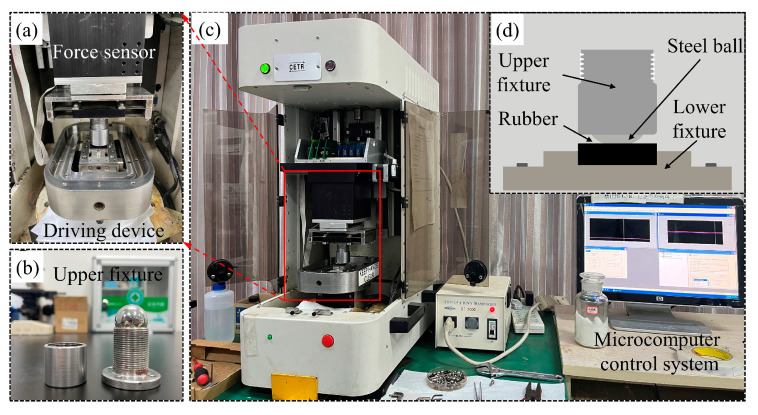
UMT-3 friction and wear tester: (**a**) friction and wear module; (**b**) upper fixture and steel balls; (**c**) friction and wear tester; and (**d**) reciprocating friction principle.

The friction and wear tests under actual well fluid lubrication conditions of high-acrylonitrile rubber after thermal expansion and swelling were conducted using the UMT-3 friction and wear tester (Figure 5a). The reciprocating sliding method of ball/surface was employed (Figure 5b), with a 304 stainless steel ball of 25 mm in diameter as the upper test specimen (Figure 5c). This steel ball made reciprocating movements on the surface of the rubber (the lower test specimen) (Figure 5d). The upper specimen was secured on the testing machine using a self-designed fixture, while the lower specimen was held in place by a rectangular slot fixture attached to a driven working platform. The friction force was transmitted to the control microcomputer using a force transducer. The UMT-3 friction and wear tester was set up in the form of reciprocating friction with an applied load of 50 N, a reciprocating frequency of 3 Hz, a reciprocating stroke of 6 mm, and a friction time of 2400 s. The friction force was transmitted to the control microcomputer using a force transducer.

## 3. Results and Discussion

### 3.1. Analysis of High-Acrylonitrile Rubber Swelling Property Test Results

**Figure 6:**
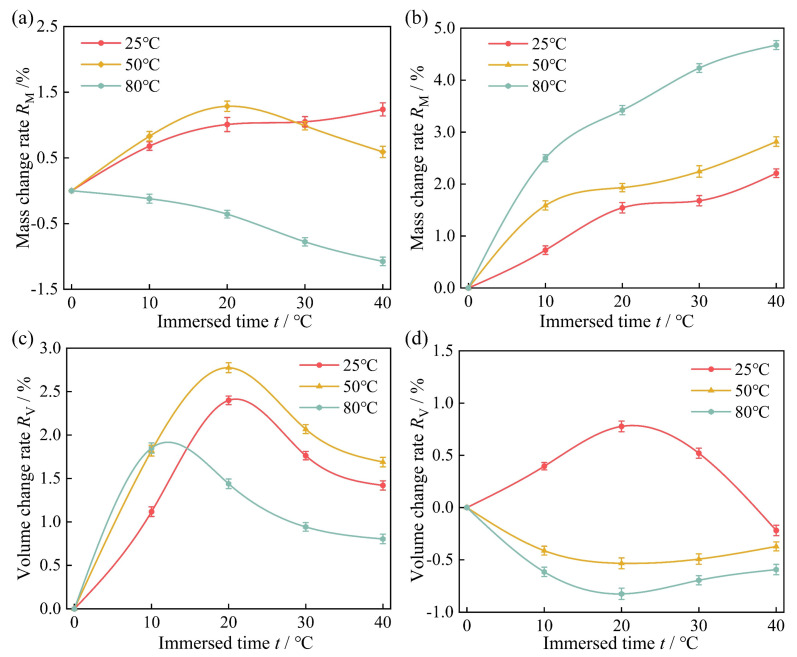
Mass and volume change rates of high−acrylonitrile rubber immersed in actual well fluids and diesel oil at 25 °C, 50 °C, and 80 °C across different times: (**a**,**c**) actual well fluids; (**b**,**d**) diesel oil.

In this study, Figure 6 illustrates the mass change and volume change rates of high-acrylonitrile rubber immersed in actual well fluids and diesel oil at 25 °C, 50 °C, and 80 °C for different times. Figure 6a demonstrates that, with increasing immersion time, the mass change rate of the rubber mass increased gradually under the condition of 25 °C. Under the condition of 50 °C, the mass change rate initially increased, peaking on the 20th day, and then decreased. At 80 °C, the mass change rate consistently decreased over time. According to the analysis, the rubber specimen exhibits two behaviors—thermal expansion and swelling—as well as molecular precipitation during these processes. The early stages of rubber specimen immersion at 25 and 50 °C show an increase in mass change rate that is directly correlated with the rubber specimens’ absorption of molecules in the immersion solution. The quality of the rubber specimen improves as more of the immersion media penetrates it. Rubber molecule precipitation increases with immersion time and contributes significantly to the rubber samples’ decreased mass change rate. The rubber’s mass change rate continuously drops at 80 °C, a phenomenon linked to the rubber’s molecular chain breaking and rubber molecule precipitation.

In Figure 6b, it was observed that in diesel oil, the mass of the rubber specimens increased with immersion time at all three temperatures. The rate of increase was higher at elevated temperatures. This was because the increase in temperature led to a decrease in the viscosity of the immersion medium, enhancing the mobility and kinetic energy of its molecules [49,50]. Thus, the medium was more likely to penetrate the rubber [51]. Simultaneously, the increase in temperature led to an increased rubber molecular chain and molecular gap, facilitating easier penetration of the medium molecules into the rubber [52].

Figure 6c indicates that, with increased immersion time, the volume change rate of the high-acrylonitrile rubber increased initially and then decreased in all three temperatures of the actual well fluid. This was associated with rubber specimens absorbing the immersion medium and rubber’s changing molecular structure. Rubber specimens absorbing immersion medium molecules caused volume expansion; as immersion time increased, the rubber’s molecular chains became more severely broken and crosslinked, reducing volume. Under the conditions of 25 °C and 50 °C, the volume change rate of the rubber peaked on the 20th day and the 10th day at 80 °C. Figure 6d indicated that in diesel oil, the rubber volume change rate of high-acrylonitrile was within ±1% across all three temperatures. The volume change pattern varied with temperature. Under 25 °C working conditions, it first increased and then decreased, while at 50 °C and 80 °C, the volume change rate was negative. The negative volume change rate at higher temperatures was attributable to excessive cross-linking of the molecular network structure of the rubber and the precipitation of molecules within the rubber.

Furthermore, both the rubber mass and volume in the actual well fluid and diesel oil changed more rapidly during the initial 10 days of the test. At 25 °C and 50 °C, the rubber specimens in both media reached a stable state of swelling and dissolution on the 30th day. The increased temperature resulted in the internal molecular chains of the high-acrylonitrile rubber breaking and cross-linking, which destroyed the molecular network structure, leading to the precipitation of some rubber molecules [35]. This resulted in a reduction in mass, leading to a change in mass of the rubber specimen in the actual well fluid at 80 °C to be negative.

According to the screw pump swelling detection standard specification, a rubber swelling value below 5.0% is normal, 5.0–7.5% serves as a warning threshold, and values >7.5% are strictly prohibited. In this study, the maximum mass change rate of high-acrylonitrile rubber in 80 °C diesel oil was 4.68%, and the maximum volume change rate in 50 °C actual well fluid was 2.78%. Both maximum mass and volume change rates were <5.0%, indicating that the high-acrylonitrile rubber utilized in this study exhibited superior resistance to swelling under the tested conditions.

### 3.2. Analysis of High-Acrylonitrile Rubber Mechanical Property Test Results

**Figure 7:**
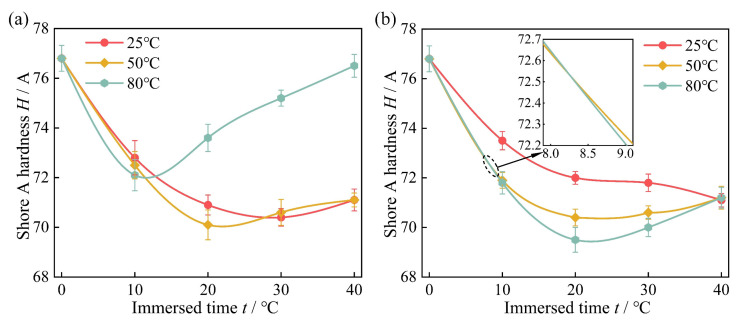
Shore A hardness change of high-acrylonitrile rubber immersed in the actual well fluid and diesel oil at 25 °C, 50 °C, and 80 °C for different times: (**a**) actual well fluid (**b**) diesel oil.

Figure 7 shows the shore A hardness of high-acrylonitrile rubber immersed in actual well fluids and diesel oil across different times at 25 °C, 50 °C, and 80 °C. Figure 7a illustrates that the rubber hardness initially decreased and then increased with increasing immersion time in actual well fluids. At 25 °C, the rubber hardness was lowest on the 30th day. At 50 °C, the hardness was lowest on the 20th day. At 80 °C, the hardness was lowest on the 10th day. The minimum hardness values at these temperatures in the actual well fluids were 70.4, 70.6, and 72.1. Figure 7b shows that with the extension of immersion time in diesel oil, the rubber hardness decreased at 25 °C; the hardness change was more stable after 20 days, with a minimum value of 71.1 on the 40th day. At 50 °C and 80 °C, the hardness decreased initially and then increased, reaching minimum values of 70.6 and 70 on the 20th day, respectively.

Given the high-acrylonitrile rubber in the two media for the thermal expansion and swelling test, it was observed that in the first 10 days of the test, the rubber hardness decreased rapidly. Rubber hardness decreased with increasing temperature. In actual well fluid and diesel oil at 80 °C, the hardness reduction rates reached 6.1% and 6.5%, respectively. This initial decrease was attributable to thermal expansion and swelling, where the rubber absorbed molecules from the immersion medium, resulting in a decreased molecular density and hardness. Subsequently, with increasing immersion time, the molecular chain inside the rubber was destroyed by the small molecules of the immersion medium, and the molecular chain underwent cross-linking and rupture reactions, which eventually increased the hardness and brittleness of the rubber [53,54].

**Figure 8:**
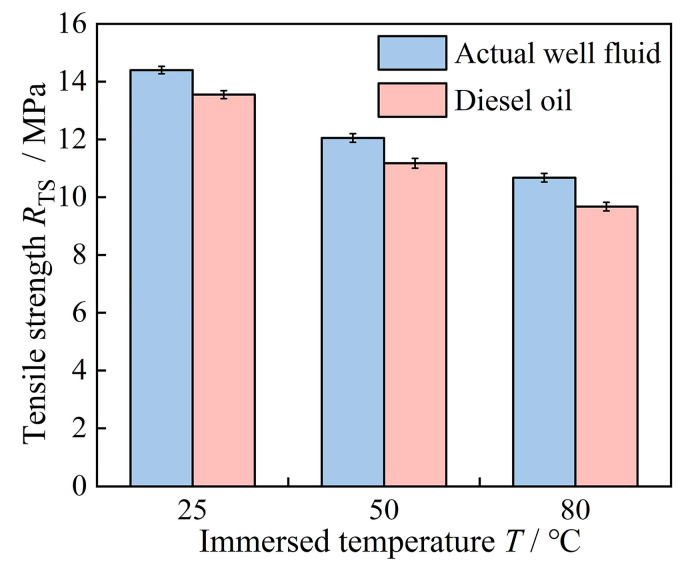
Tensile strength of high-acrylonitrile rubber after 40 days of immersion in actual well fluids and diesel oil at 25 °C, 50 °C, and 80 °C.

Table 1 and Figure 8 present the elongation at break and tensile strength of high-acrylonitrile rubber after 40 days of immersion in actual well fluids and diesel oil at 25 °C, 50 °C, and 80 °C. From Table 1, it was observed that temperature significantly influenced the elongation at break of high-acrylonitrile rubber, with the smallest elongation at 80 °C. The elongation at break after 40 days of immersion in actual well fluid and diesel oil at 80 °C was 358% and 338%, respectively, with the rubber elongation at break in diesel oil decreasing by over 50%. The decrease in elongation at a break of the high-acrylonitrile rubber specimens in diesel oil was greater compared with the actual well fluid across all three temperatures.

By analyzing the fracture of the rubber specimens after tensile fracture, it was found that there were cracks extending from the rubber surface to the inside of the fracture. The reason was that the surface and subsurface molecular chains of rubber are broken after thermal expansion and swelling. After the immersion medium entered the rubber, the rubber mesh structure expanded and deformed; some physical crosslinking points broke; cracks and holes appeared in the network structure; and finally, the mechanical properties of the rubber were significantly reduced. Considering the aging of rubber at the same time, it was considered that the tensile fracture mode of the rubber specimen was brittle fracture.

Figure 8 indicates that the change in tensile strength of high-acrylonitrile rubber correlated with the change in elongation at break. The tensile strength decrease became evident with increasing temperature. After 40 days of immersion at 80 °C, the tensile strength decreased to 10.7 MPa and 9.7 MPa in actual well fluid and diesel oil, respectively. The elongation at break and tensile strength of rubber are primarily affected by the molecular chain, and increasing immersion temperature led to increased molecular chain fractures, reducing both elongation at break and tensile strength.

### 3.3. Microanalysis of Rubber Surfaces after Thermal Expansion and Swelling

**Figure 9:**
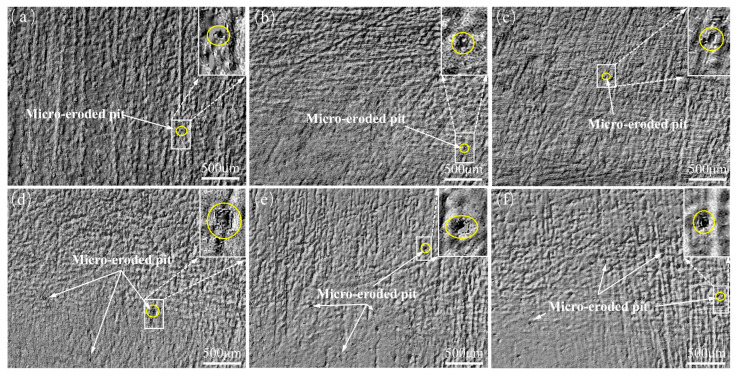
Surface morphology of high-acrylonitrile rubber after 40 days of immersion: (**a**) 25 °C, actual well fluid; (**b**) 50 °C, actual well fluid; (**c**) 80 °C, actual well fluid; (**d**) 25 °C, diesel oil; (**e**) 50 °C, diesel oil; (**f**) 80 °C, diesel oil.

Figure 9 depicts the surface micro-morphology of high-acrylonitrile rubber after 40 days of immersion in actual well fluids and diesel oil at 25 °C, 50 °C, and 80 °C. Furthermore, due to prolonged exposure to the soaking medium, the rubber surface underwent erosion, allowing the medium molecules to permeate the rubber, disrupting its three-dimensional cross-linking network. The result was molecular chain breakage and the extraction of internal substances from the rubber. After thermal expansion and swelling, the rubber material became less rigid and softened, leading to the emergence of micro-eroded pits and bulges across all surfaces of the high-acrylonitrile rubber. At the same time, there was also a sulfide layer that was not eroded by medium molecules.

### 3.4. Analysis of High-Acrylonitrile Rubber Friction and Wear Property Test Results

**Figure 10:**
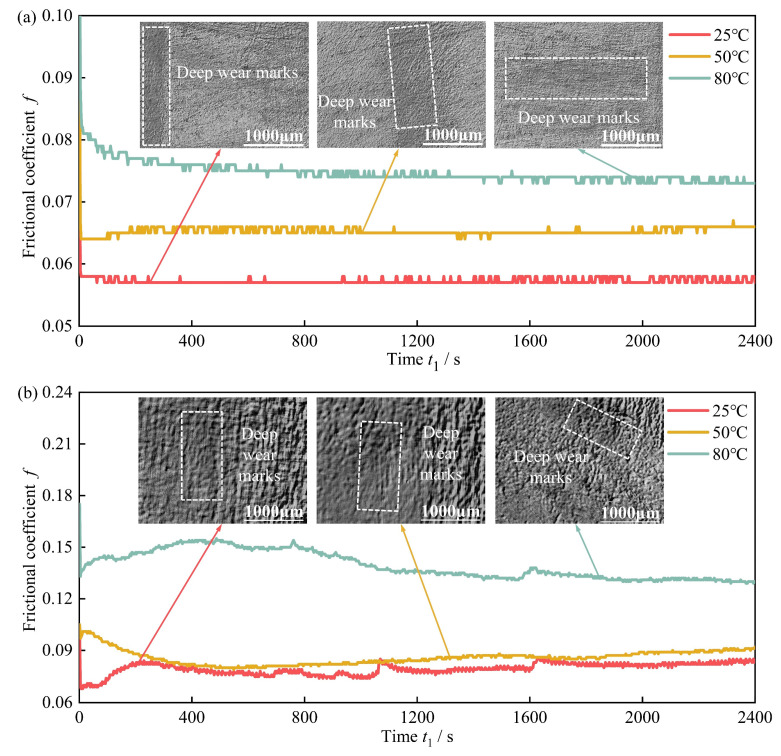
Surface morphology of high-acrylonitrile rubber after 40 days of immersion: (**a**) actual well fluid and (**b**) diesel oil.

**Figure 11:**
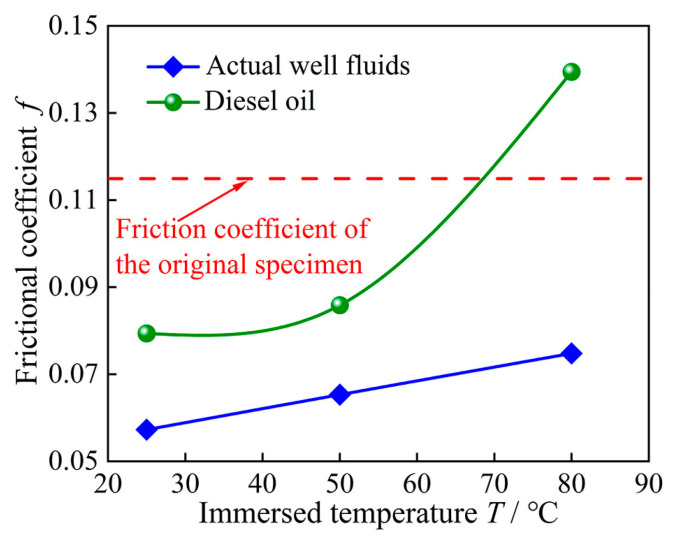
Average friction coefficient of rubber surfaces after 40 days of immersion at different temperatures.

Table 2 presents the mean and standard deviation of the average friction coefficients and wear quantities for each set of rubber specimens after friction and wear testing. Figure 10 and Figure 11 depict the friction coefficient curves, rubber surface wear micro-morphology, and average friction coefficient of high-acrylonitrile rubbers after 40 days of thermal expansion and swelling in actual well fluids and diesel oil at three temperatures. Both actual well fluids and diesel oil exhibited an increase in the surface friction coefficient of rubber specimens with increasing immersion temperatures. The abrasion marks became more evident, with a significant increase in abrasion observed at 80 °C. This phenomenon was attributable to the decrease in the modulus of rubber elasticity with increasing temperature, increasing the viscous parameter. Consequently, higher friction yielded a larger coefficient of friction. As shown in Figure 11, compared with the rubber without thermal expansion and swelling, the average friction coefficient of the rubber decreased in actual well fluid as well as in diesel oil at 25 °C and 50 °C. However, the average friction coefficient increased for rubber immersed in diesel oil at 80 °C.

**Figure 12:**
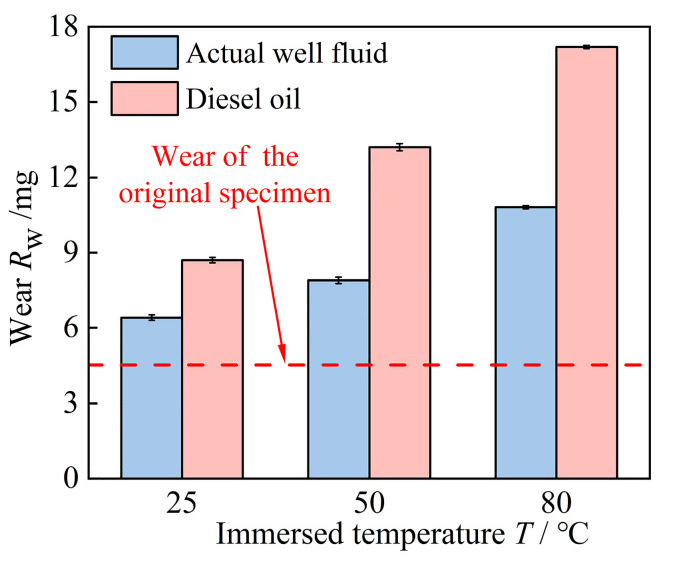
Wear of rubber surfaces after 40 days of immersion at different temperatures.

Figure 12 illustrates the wear quantity of rubber worn after 40 days of immersion at various temperatures. The wear quantity of the rubber specimens after thermal expansion and swelling increased with increasing immersion temperatures, surpassing that of high-acrylonitrile rubber without thermal expansion and swelling. This was because increased temperature induced significant aging of the rubber, resulting in decreased tear strength and increased abrasion. Temperature significantly affected the properties of high-acrylonitrile stator rubber, with higher temperatures leading to a greater decrease in rubber wear resistance. The average friction coefficient, wear amount, and abrasion marks on the rubber samples immersed in diesel oil at the same temperature for 40 days were higher compared with those immersed solely in actual well fluid. Diesel oil resulted in more damage to the high-acrylonitrile stator rubber compared with actual well fluid, significantly reducing the wear resistance of the rubber.

## 4. Conclusions

(1) With increasing thermal expansion and swelling time, the mass, volume, and hardness of high-acrylonitrile stator rubber underwent fast changes over the first ten days and then became slow. The elongation at break and tensile strength of rubber significantly reduced, and the surface damage became more serious due to the penetration of the immersion medium on the rubber surface.
(2) High-acrylonitrile rubber exhibited both thermal expansion and swelling behaviors, along with cross-linking and breaking reactions of molecular chains, when immersed to both actual well fluids and diesel oil. Thermal expansion and swelling led to increased mass and volume, accompanied by a decrease in hardness. Conversely, cross-linking, chain breakage, and precipitation of rubber molecules led to decreases in mass and volume, elongation at break, and tensile strength, coupled with an increase in hardness.
(3) Temperature significantly impacted the comprehensive properties of the rubber. An increase in temperature shifted the inflection points of the mass and volume change rate curves and hardness change curves. At higher temperatures, the rate of change of mass and volume was negative.
(4) High-acrylonitrile rubber exhibited an altered average friction coefficient after immersion in actual well fluid and diesel oil. The friction coefficient decreased after immersion in actual well fluid under the condition of actual well fluid lubrication but increased after immersion in diesel oil at 80 °C. Thermal expansion and swelling contributed to increased wear compared with the original specimens. The friction coefficient and wear quantity increased with increasing immersion temperature.
(5) The effect of diesel oil on rubber properties exceeded that of actual well fluid, with a substantial decrease in rubber tensile properties in diesel oil compared with the same temperature conditions in actual wall fluid. The wear quantity after temperature expansion and swelling was 1.36, 1.67, and 1.59 times higher in diesel oil compared with actual well fluid for the respective temperature conditions.

## Figures and Tables

**Table 1:** Elongation at break of high-acrylonitrile rubber after 40 days of immersion in actual well fluids and diesel oil at 25 °C, 50 °C, and 80 °C.

Immersion Temperature *T*/°C	Actual Well Fluid		Diesel Oil	
	Mean Value	Standard Deviation	Mean Value	Standard Deviation
25	676%	6.47%	659%	7.17%
50	455%	8.92%	444%	6.32%
80	358%	5.73%	338%	7.78%

**Table 2:** The mean values and standard deviations of the average friction coefficients of rubber samples under different thermal expansion and swelling conditions.

Thermal Expansion and Swelling Conditions of Rubber Specimens	Mean Value	Standard Deviation
Non-immersion	0.1147	6.603 × 10^−5^
Actual well fluid at 25 °C	0.0573	6.78 × 10^−5^
Actual well fluid at 50 °C	0.0653	4.626 × 10^−5^
Actual well fluid at 80 °C	0.0748	3.742 × 10^−5^
Diesel oil at 25 °C	0.0794	3.286 × 10^−5^
Diesel oil at 50 °C	0.0858	1.34 × 10^−4^
Diesel oil at 80 °C	0.1394	8.32 × 10^−4^

## Data Availability

Data available on request due to privacy reason.
